# Adaptive neuro-fuzzy inference system (ANFIS) and multiple linear regression (MLR) modelling of Cu, Cd, and Pb adsorption onto tropical soils

**DOI:** 10.1007/s11356-022-24296-8

**Published:** 2022-11-28

**Authors:** Babatunde Kazeem Agbaogun, Bamidele Iromidayo Olu-Owolabi, Henning Buddenbaum, Klaus Fischer

**Affiliations:** 1 Analytical and Ecological Chemistry, University of Trier, Trier, Germany; 2 Analytical/Environmental Unit, Department of Chemistry, University of Ibadan, Ibadan, Nigeria; 3 Remote Sensing and Geoinformatics, University of Trier, Trier, Germany

**Keywords:** Tropical soils, Heavy metals, Adsorption isotherm, Soil parameters, Modelling, ANFIS

## Abstract

Soils interact in many ways with metal ions thereby modifying their mobility, phase distribution, plant availability, speciation, and so on. The most prominent of such interactions is sorption. In this study, we investigated the sorption of Pb, Cd, and Cu in five natural soils of Nigerian origin. A relatively sparsely used method of modelling soil-metal ion adsorption, i.e. adaptive neuro-fuzzy inference system (ANFIS), was applied comparatively with multiple linear regression (MLR) models. The isotherms were well described by Freundlich and Langmuir equations (*R*^2^ ≥ 0.95) and the kinetics by nonlinear two-stage kinetic model, TSKM (*R*^2^ ≥ 0.81). Based on the values delivered by the Langmuir equation, the maximum adsorption capacities (*Q*_m_*) were found to be in the ranges 10,000–20,000, 12,500–50,000, and 4929–35,037 µmol kg^−1^ for Cd, Cu, and Pb, respectively. The study revealed significant correlations between *Q*_m_* and routinely determined soil parameters such as soil organic carbon (*C*_org_), cation exchange capacity (CEC), amorphous Fe and Mn oxides, and percentage clay content. These soil parameters, combined with operational variables (i.e. solution/soil pH, initial metal concentration (*C*_o_), and temperature), were used as input vectors in ANFIS and MLR models to predict the adsorption capacities (*Q*_e_) of the soil-metal ion systems. A total of 255 different ANFIS and 255 different MLR architectures/models were developed and compared based on three performance metrics: MAE (mean absolute error), RMSE (root mean square errors), and *R*^2^ (coefficient of determination). The best ANFIS returned *MAE*_test_ 0.134, *RMSE*_test_ 0.164, and *R*^2^_test_ 0.76, while the best MLR returned *MAE*_test_ 0.158, *RMSE*_test_ 0.199, and *R*^2^_test_ 0.66, indicating the predictive advantage of ANFIS over MLR. Thus, ANFIS can fairly accurately predict the adsorption capacity and/or distribution coefficient of a soil-metal ion system a priori. Nevertheless, more investigation is required to further confirm the robustness/generalisation of the proposed ANFIS.

**Supplementary Information:**

The online version contains supplementary material available at 10.1007/s11356-022-24296-8.

## Introduction

Naturally, metals are present in soils as trace elements from parent materials, and most of them are essentials for animal and plant metabolism (Silveira et al. 2003). However, anthropogenic activities such as discharge of industrial effluents and mine tailings; open dumping of untreated solid waste in lands designated as dumpsites and/or in unlined landfills, a common practice in developing economies; and sewage sludge application on agricultural and forested land (especially in the developed economies) have been linked to serious spikes in the natural concentrations. This has posed a great stress on groundwater and freshwater as well as food resources, with concomitant consequences on health of human beings and other ecological receptors. A recent case of heavy metal poisoning was witnessed in Zamfara state, Northwestern Nigeria, where over four hundred children reportedly died within the first few months of lead pollution derived from illegal artisanal gold mining (Biya et al. 2010; Blacksmith Institute 2010; Greig et al. 2014).

Due to their non-degradability, metal ions cannot be transmuted/mineralised to totally innocuous forms. Previously, environmental quality assessment for heavy metals in soils was based on total concentrations, but a strong argument now exists for basing it on metals in solution, i.e. the labile fraction instead. This is due to the observation that transportation of metals from soils into the freshwater ecosystem is dependent on their presence in the solution phase (Rieuwerts et al. 1998). However, nature, in particular soils, interacts in many different ways with this labile fraction, thereby modifying metal mobility, phase distribution, bioavailability, speciation, toxicity, and so on. The most prominent of such interactions are sorption-cation exchange, complexation, and specific adsorption (Strawn 2021). Sorption has long presented great interests to both environmental and soil scientists (Dube et al. 2001; Strawn 2021).

Investigating the sorption behaviour of heavy metals in soils not only gives vital information about their environmental risk, but also provides useful knowledge for remediation of heavy metal–contaminated soils/sites. The conventional method of carrying out sorption tests is by field or laboratory experiments. However, not only are these experiments laborious and expensive, they could also be fraught with many errors. Furthermore, mostly, the adsorption capacity of soils for heavy metal cations is controlled by soil texture; soil composition, especially the composition and amount of the mineral fractions (i.e. metal hydr-(oxides) and clay); the amount and property of soil organic matter; soil pH; cation exchange capacity; and other reaction variables, all of which can interact (Guanshu and Baoshan 2001; Katseanes et al. 2016; Agbaogun and Fischer 2020). Thus, variations in environmental fate parameters such as partition coefficients are commonly attributed to differences in soil physicochemical properties (Katseanes et al. 2016). Therefore, with the high level of spatial variations in soils and soil attributes alone, it is scientifically inconceivable to investigate all soil-metal adsorption systems. This makes sorption of metal ions by soils, just like that of any other chemical contaminants, a complex process that may be very difficult to formalise by means of conventional statistical/mathematical methods, hence the need for simple and effective estimation/prediction methods.

**Fig. 1:**
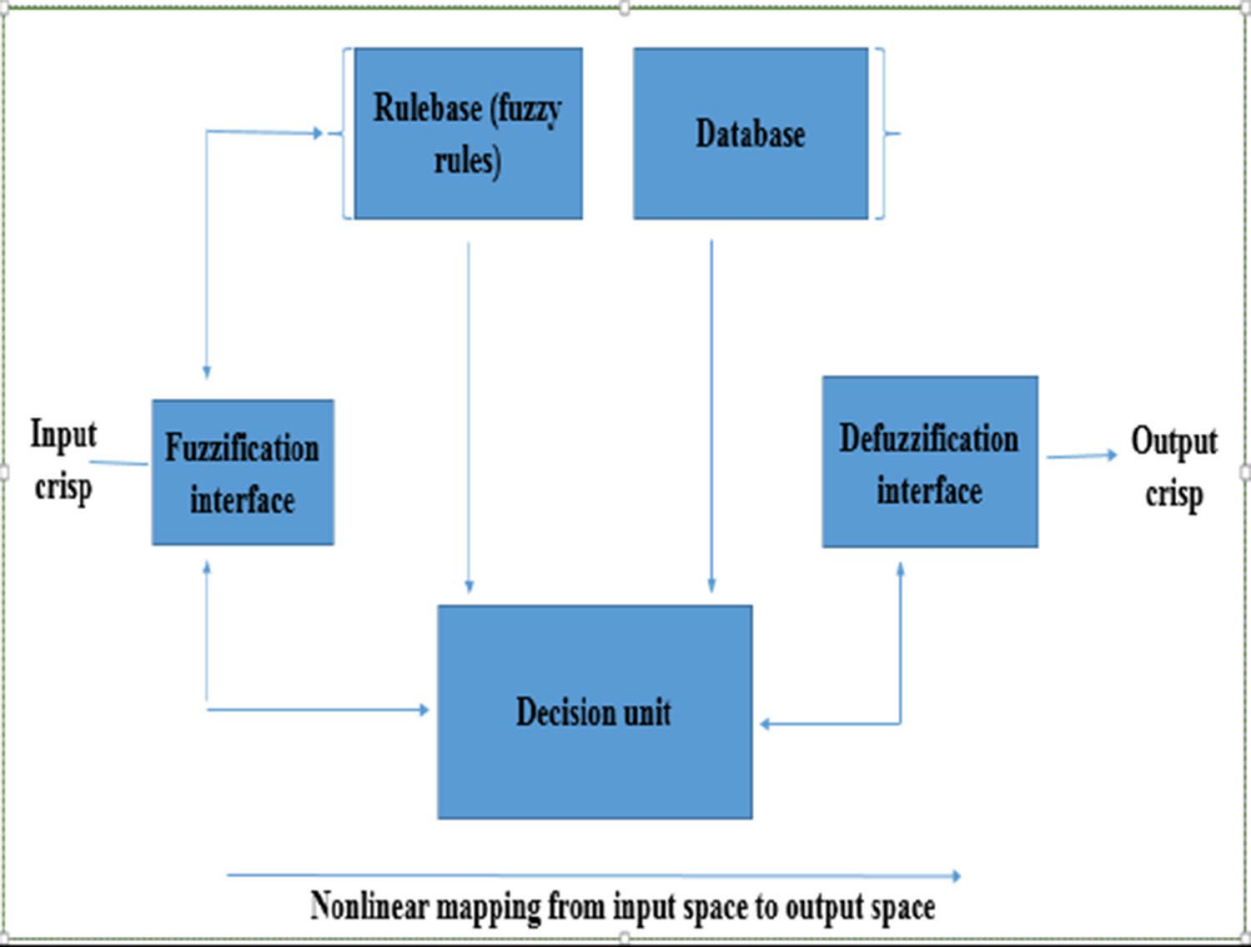
Schematics of a fuzzy inference process with crisp output

Recently, soft computing methods such as artificial neural network (ANN), fuzzy logic (FL)–based techniques (i.e. fuzzy inference systems, FIS), genetic algorithms (GA), and several connectionist systems such as adapted neuro-fuzzy inference system (ANFIS) are increasingly being recognised as accurate learning schemes for modelling complex phenomena in different aspects of engineering, physical, and natural sciences (Agbaogun et al. 2021). These techniques have the capability to take care of uncertainties that often accumulate in traditional mathematical and statistical techniques (Kebria et al. 2018). ANFIS is a hybrid of ANN and FIS. ANN is an advanced mathematical tool that is inspired by the biological neural structure of the brain (Souza et al, 2018). In analogy with human brain, ANN consists of single units (neurons) that are interconnected by the so-called synapses (Dolatabadi et al, 2018). ANN has the ability to learn from an input and output pair of data and adapt to it in an interactive way (Tiwari et al. 2018). FIS, on the other hand, is inspired by fuzzy logic (FL) which is a heuristic system description that uses “if–then” rules to establish quantitative relationships among the input and output vectors (Vernieuwe et al. 2005). FL is based on fuzzy set rules. Broadly defined by Zadeh (1965), a fuzzy set is a class of objects with continuum of grades of “membership”; that is, every object is assigned a condition of membership ranging between zero and one. Fuzzy sets rely on FL operations and parallel if–then rules to execute a nonlinear mapping of an input space to the output space through membership functions (Fig. 1) to form the fuzzy inference system. In other words, ANFIS is a kind of ANN that is based on Takagi–Sugeno Kang (TSK) fuzzy inference system (Jang 1993). This inference system has learning capability to approximate nonlinear functions (Aqil et al. 2007) and particularly gaining popularity in dealing with ill-defined and uncertain domains; hence, it is considered a “universal estimator”. Further, it is well-known for its ability to produce systems with good interpretability-accuracy trade-off by combining the advantages of ANN and FIS in a single framework and equally reduces the drawbacks of the two (Rahimzadeh et al. 2016; Agbaogun et al. 2021).

Apart from numerous near-field applications, a copious number of articles where ANFIS was used in predicting the adsorption of heavy metal ions and/or organic chemical contaminants onto several adsorbents have surfaced in the literature, especially in the last one decade. These include, but not limited to, Qasaimeh et al. (2012), Amiri et al. (2013), Ghaedi et al. (2013, 2014), Tanhaei et al. (2017), Baziar et al. (2017), Dolatabadi et al. (2018), Lashkenari et al. (2018), Javadian et al. (2018), and Sigmund et al. (2020). Most recently too, Agbaogun et al. (2021) successfully used ANFIS to model the adsorption of phenylurea herbicides by soils. So far, our literature search has revealed that ANFIS has never been used in modelling the adsorption of heavy metal ions onto natural soils.

Therefore, apart from contributing to the body of knowledge on the sorption behaviour of Pb, Cu, and Cd in Nigerian (tropical) soils, this paper will be more concerned with the search for the subset(s) of variables that can best predict soil adsorption capacities (*Q*_e_) for these metal ions. Thus, just like in our previous similar studies on modelling of soil organic contaminant phase distribution, the major questions this paper will be answering are (i) since literature is replete with reports of significant correlations between some routinely measured soil attributes and metal ion phase distribution, is a combination of such soil attributes sufficient as a pedotransfer function for in silico estimation of soil-metal ion adsorption coefficients, (2) what are the major drivers for soil-metal ion adsorption, and (3) can a simple traditional system like MLR predict these phase distributions in soils more accurately than an advanced expert system like ANFIS? As earlier stated, ANFIS, although widely reported for their robustness in extracting complex nonlinear relationship from data, has never been applied to modelling the partition coefficients of metal ions in soil. This work will be the first to integrate experimental dataset in expert systems like ANFIS to develop a capability for predicting the phase distribution of these chemical species in soils, hence the novelty of this work. It is worth noting that in this paper, we search not only for an accurate prediction, but we research for the smallest combination of inputs to produce such prediction. We also evaluated the sensitivity of the models to each of the regressed vectors. Accordingly, we produce a robust and trustworthy model with a good interpretability-accuracy trade-off.

## Materials and methods

### Soils characterisation

Five topsoil samples were selected from a pool of soils stemming from southwestern Nigeria. This region is covered with dense forest and savannah vegetation (trees and shrubs), with and without canopy formation (Fagbemi and Shogunle 1995). The climate is characterized by 28–32 °C (annual average) temperature and a mean annual precipitation of 1000–1500 mm, with the rainy season lasting for 7–8 months. Generally, the soils are ferruginous tropical soils with kaolinite as the dominant clay mineral (Agbaogun and Fischer, 2020). According to US soil taxonomy, the dominant soil types in this region are Arenic Paleudalfs, Rhodic Paleudalfs, Oxic Tropudalfs, Typic Tropudults, Typic Tropaquepts, Oxic Paleudalfs, Oxic Paleustalfs, Aquic Tropopsamments, and Typic Ustipsamments (pro parte) (Fagbemi and Shogunle 1995). These can be broadly classified as Luvisols, Lixisols, Gleysols, and Arenosols according to the IUSS Working Group WRB (2014). As observed by Giresse (2008), almost all the tropical soils are fairly represented in the Western part of the African continent, hence the choice of this study location. The soils were selected to cover a relatively wide range of physicochemical parameters. The physicochemical properties of the soils were determined as previously described by Agbaogun and Fischer (2020).

### Metal ion solutions

Two thousand–mg/L Pb^2+^, Cu^2+^, and Cd^2+^ stock solutions were prepared from lead (II) nitrate (Pb(NO_3_)_2_), copper (II) nitrate hemipentahydrate (Cu(NO_3_)_2_.2.5H_2_O), and cadmium (II) nitrate tetrahydrate (Cd(NO_3_)_2_.4H_2_O), respectively. Working solutions in the range 10–180 mg/L were prepared by serial dilution of the stock in 0.001-M KNO_3_ (indifferent electrolyte). Nitrate was chosen as indifferent electrolyte because of its small capacity for complexation with cations (Msaky and Calvet 1990; Silveira et al. 2003). Other chemicals used include nitric acid (HNO_3_) and sodium hydroxide (NaOH) which were received in analytical quality from Sigma Aldrich, Germany.

### Adsorption experiments

For sorption kinetics tests, 1 g each of the samples, except for UI (Uni-Ibadan) that was 0.5 g, was mixed with 50 mL of 50-mg L^−1^ solution of the metal ions in 100-mL polypropylene centrifuge tubes. Samples were withdrawn at 15 min, 30 min, 60 min, 180 min, 360 min, 540 min, 720 min, and 1440 min of contact. For the effects of pH, 1 g of the samples was equilibrated for 24 h with 20 mL (except for UI that was 30 mL) of 50-mg L^−1^ pH conditioned metal ion solutions. The solutions were conditioned to pH 2.0 ± 0.1, 3.0 ± 0.1, 4.0 ± 0.1, 5.0 ± 0.1, 6.0 ± 0.1, and 7.0 ± 0.1 with either dilute HNO_3_ or NaOH solution. Adsorption isotherms and effects of concentrations tests were carried out in a thermostated rotary shaker for 24 h at 293 K, 313 K, and 333 K, with 1 g each of the samples (except for UI-Pb that was 0.5 g) and 20 mL of solutions with metal ion concentrations ranging from 10 to 60 mg L^−1^ (for AK-Pb at 293 K, BK-Pb at 293 K, and for IB-Pb and OD-Pb, at the three temperatures). AK, BK, IB, and OD here represent the sampling locations Akanran, Bakatari, Ibadan, and Odeda, respectively. Other isotherm tests were carried out with metal ion concentrations ranging from 30 to 180 mg/L. At the end of the reaction period, the mixtures were centrifuged at 3000 rpm for 15 min. The supernatants were then filtered with 0.45-µm regenerated cellulose (RC) membrane filters (Millipore, VWR, Germany), and the filtrates were analysed for their residual metal ion concentrations with atomic absorption spectrometry, AAS (Varian AA240FS, Varian Inc., Germany). All experiments were performed in duplicate. Blank samples and controls were also run in parallel for quality control measures. It was assumed that the differences between the initial metal ion concentrations (*C*_o_, µmol L^−1^) and the residual concentrations in the aqueous phase (*C*_e_, µmol L^−1^) were solely due to sorption. Therefore, the amount adsorbed by soil (*Q*_e_, µmol kg^−1^) was calculated based on mass balance as follows:

1 $${Q}_{e}=\left(\frac{{C}_{o}-{C}_{e}}{{m}_{s}}\right)*V$$

where *V* (L) is the volume of the solution and *m*_s_ is the mass of the soil (kg). The linear distribution coefficient (*K*_d_) was calculated from Eq. 2:

2 $${K}_{d}= \frac{{Q}_{e}}{{C}_{e}}$$

Stemming from two traditional Eqs. 1 and 2,

3 $${K}_{d}= \frac{{Q}_{e}V}{{C}_{0}V- {Q}_{e}Ms}$$

For 1 L (*V*) of a specified initial metal ion concentration and 1 kg of soil (*Ms*), *K*_d_ can be calculated from the values of *Q*_e_ (experimental or predicted) by Eq. 4 (Agbaogun et al. 2021).

4 $${K}_{d}= \frac{{Q}_{e}}{{C}_{0}- {Q}_{e}}$$

Further, the adsorption mechanisms were empirically described by various mathematical equations. For sorption isotherms, two most commonly used equations—Langmuir and Freundlich (Eqs. 5 and 6)—were used to fit the isotherm data, using the nonlinear least square method. The kinetics data were fitted to pseudo second-order (PSO) equation and the two-stage kinetic model, TSKM (Eqs. 7 and 8, respectively), also using nonlinear least square method.

5 $${Q}_{e}=\frac{K{Q}_{m}^{*}{C}_{e}}{1+K{C}_{e}}$$

6 $${Q}_{e}= {K}_{f}{C}_{e}^{n}$$

7 $${Q}_{t}= \frac{{k}_{2}{Q}_{e}^{2}t}{1+ {k}_{2}{Q}_{e}t}$$

8 $${Q}_{t} \left(t\right)= {Q}_{e} \frac{t}{\frac{1+{k}_{1}t}{{k}_{1}}}+2*{k}_{2}*{t}^{0.5}$$

where *Q*_m_^*^ is the maximum adsorption capacity (µmol g^−1^) of the adsorbent, *Q*_*e*_ and *C*_e_ are as earlier described in Eq. 1, *K*_L_ (L µmol^−1^) is the Langmuir constant that is related to the affinity of the binding sites, *K*_f_ (µmol^1−n^ L^n^ kg^−1^) is the specific Freundlich constant, and *n* (dimensionless) is the Freundlich intensity parameter which indicates the magnitude of the adsorption driving force or the surface heterogeneity.* Q*_*t*_ (µmol kg^−1^) is the amount of metal adsorbed at time *t* (min); *k*_1_ (min^−1^) is the fast rate constant, while *k*_2_ (µmol kg^−1^ min^−0.5^) is the slow or diffusion rate constant.

All linear regressions were done with Microsoft Excel® (2013), while the nonlinear regressions were developed with Matlab 2019b, using the “lsqcurvefit” algorithm. The goodness of fit of the regressions was determined by the coefficient of determination (*R*^2^), Eq. 9.

9 $${R}^{2}=1- \frac{\sum_{i=1}^{n}({y}_{i}^{*}-{y)}^{2}}{\sum_{i=1}^{n}({y}_{i}^{*}-{{y}_{m})}^{2}}$$

where y* and *y* are the observed and predicted values, respectively. *y*_m_ is the mean value of y*, and *n* is the number of observations.

### Description of ANFIS

Fuzzy neural networks are connectionist systems that integrate both neural network and fuzzy logic.

ANFIS are trained as neural networks, while their structures are interpreted as a set of fuzzy rules (i.e. the Takagi–Sugeno-Kang, TSK fuzzy rules). Ostensibly, fuzzy rules are logical sentences upon which derivation can be executed (Jang 1993). The act of executing this derivation is referred to as inference process (Jang 1993). In ANFIS, the output of each rule (consequent part) is a linear combination of input variables (their preconditions) plus a constant term, while the final output is the weighted average of each rule’s output. For instance, if the FIS under consideration is of the rule base containing two if–then rules of TSK’s type, with two inputs *x* and *y*, and one output *f*, the TSK fuzzy rules will be:

10 $${R}_{1} :IF x is {A}_{1} and y is {B}_{1}, THEN {f}_{1}= {p}_{1}x+{q}_{1}y+{r}_{1}$$

11 $${R}_{2} :IF x is {A}_{2} and y is {B}_{2}, THEN {f}_{2}= {p}_{2}x+{q}_{2}y+{r}_{2}$$

where *f*_*i*_ is output and *p*_i_, *q*_i_, and *r*_i_ are the consequent parameters of the ith rule (Agbaogun et al. 2021). *A*_i_ and *B*_i_ are linguistic labels whose membership function parameters are premise parameters and are represented by fuzzy sets (Jang, 1993). Here, the inferred output y* is calculated:

12 $${y}^{*}=f= \frac{({w}_{1}{f}_{1}+{w}_{2}{f}_{2})}{{w}_{1}+ {w}_{2}}= \overline{{w }_{1}}{f}_{1}+\overline{{w }_{2}}{f}_{2}$$

**Fig. 2:**
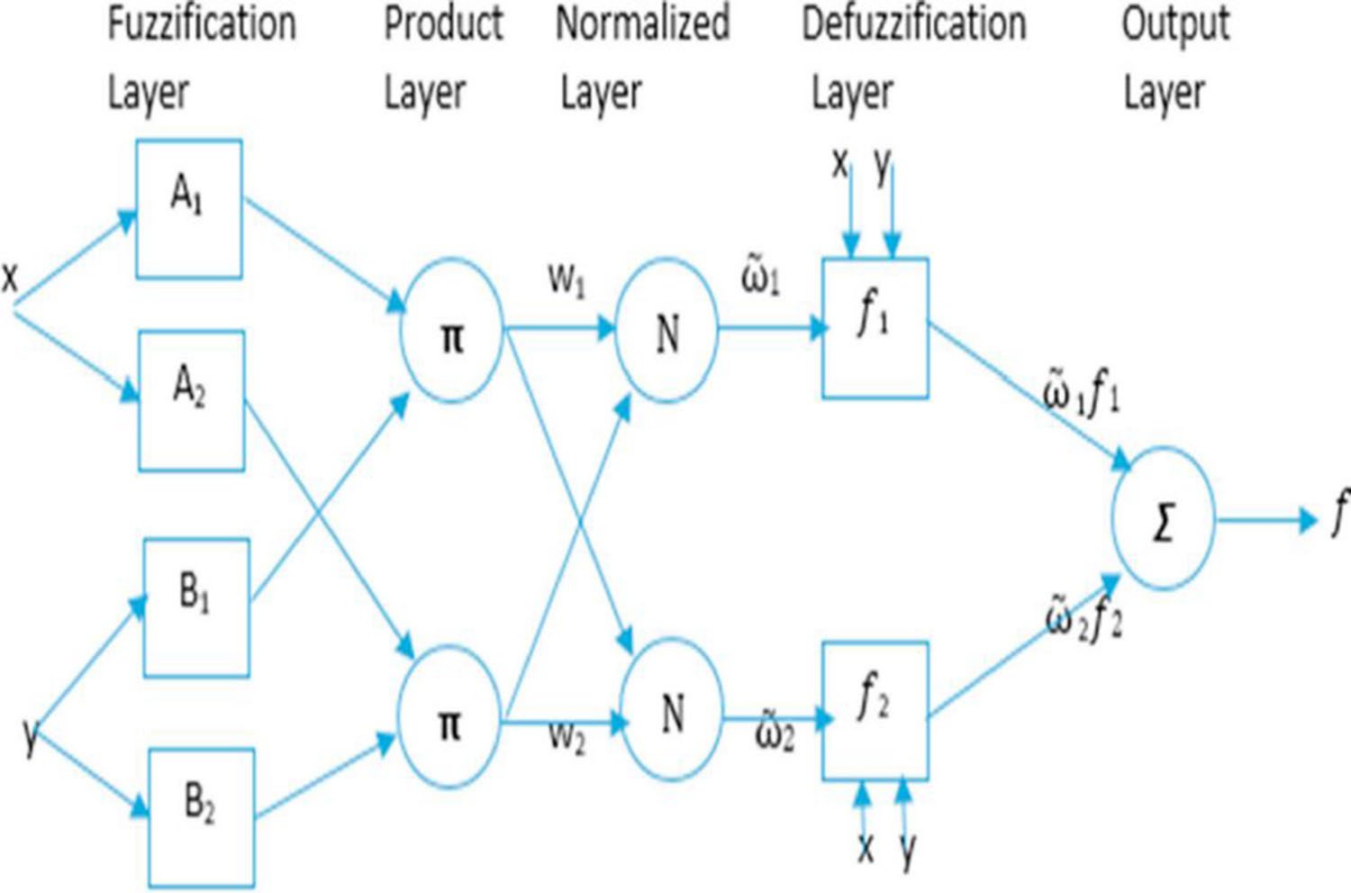
ANFIS architecture for two input vector Sugeno fuzzy systems (Jang, 1993)

Generally, an ANFIS structure consists of 5 layers: the fuzzification layer, the product layer, the normalised layer, the defuzzification layer, and the output layer (Fig. 2) (Jang, 1993). Each of these layers is tasked with different functions and contains several nodes described by the node function—i.e. adaptive nodes (for parameters that are adjustable in the system) or fixed nodes (for parameters that are nonadjustable) (Jang 1993). ANFIS training algorithm uses a combination of backpropagation gradient, descent algorithm, and a least square method to learn and recognise the pattern of the dataset (train dataset). Subsequently, another dataset (test dataset) is used to check the generalisation capability of the resulting systems. More details about the theory and applications of fuzzy set theory and the structure of ANFIS can be found in Zadeh (1965), Takagi and Sugeno (1985), and Agbaogun et al. (2021).

### MLR

Just like ANFIS, the aim of MLR is to model the relationship between the input variables and the target (or response). A time-honoured technique going back to Pearson’s 1908 use of it, MLR is employed to account for (predict) the variance in an interval dependent, based on linear combinations of interval, dichotomous, or dummy independent variables (Vatcheva et al. 2016). An MLR model with *n* observations is expressed as Eq. 13 (Brereton 2007).

13 $$y= {\varphi }_{o}+ \sum_{i=1}^{n}{\varphi }_{i} {x}_{i} + \varepsilon$$

where *y* is the dependent (predicted) variable, *φ*_o_ is the intercept, *φ*_i_ is the partial regression coefficients, *x*_i_ (*i* = 1, 2,…*n*) are predictors/independent variables, and *ε* is the random error.

MLR can be a useful predictive method, but due to its dependency on linearly correlated relationships, it may lead to inaccurate results for nonlinear and complex systems like adsorption (Yilmaz and Kaynar 2011). Again, one of the factors that affects the standard error of a partial regression coefficient is the degree to which that independent variable is correlated with the other independent variables in the regression equation (Vatcheva et al. 2016). Other things being equal, an independent variable that is very highly correlated with one or more other independent variables will have a relatively large standard error. MLR suffers the curse of multicollinearity. This is a problem because it undermines the statistical significance of an independent variable, accentuates the problem of overfitting (where the model may do well on the known training set but will fail at the unknown testing set), and reduces the precision of the estimated coefficients as well as the *p* values (Vatcheva et al. 2016).

### Modelling with ANFIS and MLR

The ambition here is to model the adsorption capacity, *Q*_e_, of the soil-metal ion system, using the batch experimental dataset. Based on the established correlations, the following eight variables were selected as potential regressors for the models: soil/solution pH, initial metal ion concentration (*C*_o_), temperature (*T*), organic carbon content (*C*_org_), cation exchange capacity (CEC), amorphous iron and manganese oxide contents (*Fe*_o_ and *Mn*_o_, respectively), and percentage clay content (%clay). These eight variables are referred to as input vectors and *Q*_e_ as the output vector. A dataset of 340 patterns was generated from these input and output vectors and was randomly divided into 90% (for training) and 10% (for testing), under tenfold cross validation. The fuzzy inference system (FIS) object was automatically generated using grid partitioning. We used the generalised bell (gbellmf) and linear membership function types for the input and output vectors, respectively. The number of membership functions for each input was set at two.

In modelling, selection of best subset of vectors is crucial in reducing the training time and improving the prediction accuracy. This is achieved by removing irrelevant, redundant, and noisy vectors (i.e. selecting the subset of vectors that can achieve the best performance in terms of accuracy, uncertainties, and explanatory power). This task is therefore one of parsimony, i.e. realising a balance between two opposing objectives: simplicity (as few regressors as possible) and fit (as many regressors as needed) (Agbaogun et al. 2021). Generally, two options are possible (i) exhaustive search (i.e. all possible regressors) or (ii) random subset of regressors. Ideally, the best subset(s) of regressors can be found by applying the exhaustive search (Al-Ani 2005), although this becomes prohibitive as the number of vectors increases. Nevertheless, we used the “all possible regressors” method in this paper since only eight input vectors were involved. Therefore, starting with one-vector models, we built the models stepwisely (i.e. sequential forward selection) until the “all eight vector” model was obtained. In this way, using Matlab 2019b, we elaborated several ANFIS and MLR models. In addition to the coefficient of determination, *R*^2^ (Eq. 8), we used two other error metrics: root mean square error (RMSE) and mean absolute error (MAE) to evaluate and compare the performances of these models. These metrics are given by Eqs. 14–15 (Chai and Draxler, 2014).

14 $$RMSE=\sqrt{\frac{1}{N}\sum_{i=1}^{n}({y}_{i}^{*}-{y)}^{2}}$$

15 $$MAE=\frac{1}{N}\sum_{i=1}^{n}\left|{y}_{i}^{*}-\right.\left.y\right|$$

where y* and *y* are the observed and predicted values, respectively, and *N* is the number of observations.

*R*^2^ gives the degree of association between predicted and measured values (Agbaogun et al, 2021). One of its useful properties is the intuitive nature of its scale (i.e. it ranges from zero to one, with zero indicating that the proposed model does not have any prediction power, while one indicates perfect prediction). RMSE indicates how close the observed data points are to the predicted values (Chai and Draxler, 2014). The lower the values of RMSE, the better the fit. MAE, on the other hand, measures the average magnitude of the errors in a set of forecasts, without considering their directions (Martin, 2020). Just like RMSE, the lower the values of MAE, the lower the prediction errors. Therefore, the best or optimal model is that which has the least MAE and RMSE and the highest *R*^2^.

## Results and discussion

### Soil characterisation

The characteristics of the soils used in this study (presented in Table 1) showed significant differences in the established components and properties related to heavy metal retention by soils. With the exception of UI which was slightly neutral (pH = 7.07), all other samples were acidic (pH 5.7–6.6). Organic carbon content varied from 4.0% in UI to 0.5% in IB and OD. The CEC ranged from 22.32 (IB) to 111.7 mmol_c_/Kg (UI). OD recorded the least cumulative pedogenic and cumulative free metal oxides (2.37 and 0.15 g kg^−1^, respectively), while UI recorded the highest of both parameters (17.37 and 4.73 g kg^−1^, respectively). UI also has the highest of clay and silt proportions (6 and 59%, respectively), while OD has the least (2.4 and 24%, respectively). Using the USDA soil texture classification, soils AK, BK, and IB were classified as sandy loam; OD was loamy sand, while UI was silty loam. The clay mineralogy revealed the presence of only kaolinite (predominantly, 77–93%) and illite.

**Table 1:**
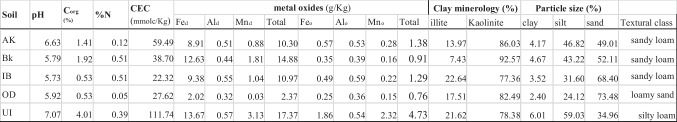
Soil physical–chemical parameters

CEC is expressed in mmol_c_ kg^−1^; subscripts “d” and “o” are dithionate and oxalate extractable metal oxides, respectively

### Adsorption isotherms

The sorption isotherm coefficients *K*_d_, *K*_f_, and *Q*_m_* are valuable parameters to compare the retention and/or interactions of metal ions with soils. High values of these coefficients indicate high retention of the metal ion by soil, while low values indicate that a large fraction of the metal remains in soil/solution, with consequences for higher mobility. Due to nonlinearity of the plots *Q*_e_ versus *C*_e_ for most of the soil-metal ion systems, *K*_d_ could not be determined as the slopes of the isotherm lines, neither could *K*_d_ at single concentration be compared because of some differences in the experimental variables. Nevertheless, the isotherm data were fitted to both Langmuir and Freundlich equations to obtain *Q*_m_* and *K*_f_, except in few soil-metal ion systems where the isotherms could not be established, and *Q*_m_* were restricted to *Q*_m_ experimental (Table 2).

**Table 2:** Sorption coefficients of the three metal ions in the soil samples at 293, 313, and 333 K

	Cd							Cu							Pb						
	Freundlich			Langmuir				Freundlich			Langmuir				Freundlich			Langmuir			
Soil/temp	*K*_f_	*n*	*R*^2^	*K*_L_	*Q*_m_*	*R*^2^	*Q*_m_	*K*_f_	*n*	*R*^2^	*K*_L_	*Q*_m_*	*R*^2^	*Q*_m_	*K*_f_	*n*	*R*^2^	*K*_L_	*Q*_m_*	*R*^2^	*Q*_m_
AK/293	1113	0.45	0.99	0.03	11,648	0.98	9216	3839	0.28	0.99	0.01	31,721	0.94	29,117	Not available						
313	2932	0.42	1.00	0.20	12,491	0.99	10,816	9564	0.20	0.97	0.05	35,412	0.96	36,720	6765	0.21	0.96	0.86	14,407	0.88	16,667
333	1373	0.47	1.00	0.05	12,581	0.99	9903	4033	0.29	0.99	0.01	32,758	0.96	31,524	Not available						16,890
BK/293	705	0.54	0.99	0.02	13,053	0.99	8953	1643	0.38	0.99	0.00	30,662	0.98	26,482	2124	0.47	0.96	0.40	6866	0.95	5398
313	2634	0.38	1.00	0.15	11,596	0.97	10,520	8197	0.20	0.99	0.05	29,955	0.92	32,390	3356	0.32	0.99	0.11	14,697	0.93	15,091
333	1137	0.48	1.00	0.04	12,083	0.97	9579	3645	0.30	0.99	0.01	30,999	0.96	30,419	5849	0.26	0.97	0.29	16,353	0.98	15,953
IB/293	402	0.53	0.98	0.01	9870	0.96	7218	2245	0.24	0.96	0.01	14,158	0.98	13,437	1674	0.35	1.00	0.27	5579	0.97	5062
313	1286	0.38	1.00	0.04	9264	0.97	8417	5189	0.14	0.95	0.03	14,530	0.94	14,423	Not available						5177
333	708	0.45	0.99	0.02	9197	0.98	7580	2727	0.23	0.98	0.01	15,426	0.95	15,188	2895	0.38	0.93	0.85	6470	0.95	5414
OD/293	390	0.52	0.99	0.01	9479	0.98	6631	2115	0.23	0.90	0.01	12,881	0.98	11,508	1566	0.33	0.95	0.23	5251	0.96	4929
313	1398	0.34	0.99	0.04	8558	0.96	7977	3878	0.17	0.92	0.01	13,515	0.98	13,139	Not available						5132
333	756	0.41	0.99	0.02	8016	0.98	7015	2287	0.24	1.00	0.01	14,592	0.93	14,418	2383	0.35	0.93	0.66	5771	0.94	5363
UI/293	2445	0.49	0.99	0.12	14,581	0.98	10,830	10,241	0.28	0.98	0.09	46,000	0.92	50,181	Not available						35,026
313	5715	0.53	0.98	0.59	16,229	0.99	11,203	Not available						55,485							35,031
333	2789	0.66	0.99	0.13	20,877	1.00	11,115	8541	0.37	0.91	0.04	61,027	0.99	52,608							35,037

*Q*_m_*, the Langmuir maximum adsorption capacity of the soil, µmol kg^−1^; *Q*_m_, experimental maximum adsorption capacity of the soil, µmol kg^−1^; *K*_L_, the Langmuir constant that is related to the affinity of the binding sites, L µmol kg^−1^; *K*_f_, Freundlich constant, µmol^1−n^ L^n^ kg^−1^; *n*, Freundlich parameter related to surface heterogeneity, dimensionless; and *R*^2^, the coefficient of determination

From the results, both equations have almost similar fits, with *R*^2^ ranging from 0.93 to 1.0. Bradl (2004) has also concluded that adsorption behaviour of heavy metals in soils can be described adequately by either Freundlich or Langmuir model (Bradl 2004). Arising from the Freundlich plots, we observed *n* ˂ 1 in all cases. While the general trends of *n* with basic soil properties were not readily discernible, however, UI with the highest %*C*_org_ (4.01) recorded the highest *n* values (i.e. 0.74 for Cd and 0.51 for Cu). Generally, given the heterogeneous nature of soil surfaces, increasing surface coverage makes less active sites accessible, thus leading to less stable surface binding and/or higher probability of desorption. The lower the value of *n*, the less stable the surface binding and/or the higher the probability of desorption.

We observed that *K*_f_ and *Q*_m_*/*Q*_m_ for the three metal ions followed the trend UI > AK > BK > IB ≥ OD. This correlates well with most of the measured soil physicochemical parameters, notably %*C*_org_, pH, CEC, total pedogenic and amorphous Fe and Mn oxides, and %clay content. For instance, UI which has the highest values of all measured soil parameters had the highest *Q*_m_* and *K*_f_, whereas IB and/or OD with the least of all the soil parameters had the least of the coefficients, thus confirming the already established soil physicochemical control of metal ion adsorption.

**Fig. 3:**
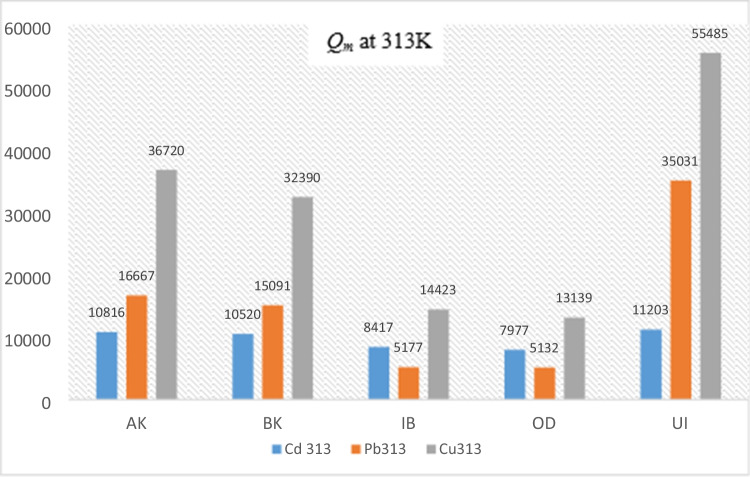
Trend of *Q*_*m*_ in the soil samples at 313 K

The trend of *Q*_m_* with the metals followed the sequence Cu ˃ Pb ˃ Cd. This correlates with the orders of their electronegativity (Pauling scale). It also correlates with respective first hydrolysis constants (*pK*_b_) of the metals, indicating that hydroxo-species (MeOH^+^) may play an important role in the formation of the surface complexes. However, apart from Cd which returned the least *k*_f_, the ion with higher *k*_f_ between Cu and Pb cannot be readily established. For Cd and Cu, *Q*_m_*/*Q*_m_ increased with increase in temperature from 293 to 313 K, but decreased from 313 to 333 K. For Pb however, *Q*_m_ increased with increase in temperature from 293 to 313 K and further increased from 313 to 333 K. Figure 3 shows the trend of *Q*_m_ of the metals in the soils at 313 K.

We also compared our results, *Q*_m_*/*Q*_m_ especially, with the values reported in the literature for soils from other tropical climes, including Nigerian soils from agroecological zones different from the present study area. In this study, the values obtained for Cu (12,881–61,027 µmol kg^−1^) is within the range (i.e. 24,700–199,200 µmol kg^−1^) reported for Cu by Sangiumsak and Punrattanasin (2014) in Thailand soils. Generally, values reported for the three metals in other tropical soils are within our ranges. For instance, Cazanga et al. (2008), Moreira and Alleoni (2009), Xie et al. (2018), and Diagboya et al. (2015) reported 38,300, 2600–31,500, 36,000, and 41,385–71,754 µmol kg^−1^ respectively for Cu in Chilean, Brazilian, Chinese, and Nigerian soils. Cazanga et al. (2008) and Diagboya et al. (2015) reported 48,100 and 33,200–72,010 µmol kg^−1^ for Pb in Chilean and Nigerian soils, respectively, while we reported 5062–35,037 µmol kg^−1^. Also, Moreira and Alleoni (2009) and Diagboya et al. (2015) reported 3500–6800 and 9074–17,881 µmol kg^−1^ for Cd in Brazilian and Nigerian soils, respectively, while this study reported 8000–21,100 µmol kg^−1^.

### pH dependency of adsorption

**Fig. 4:**
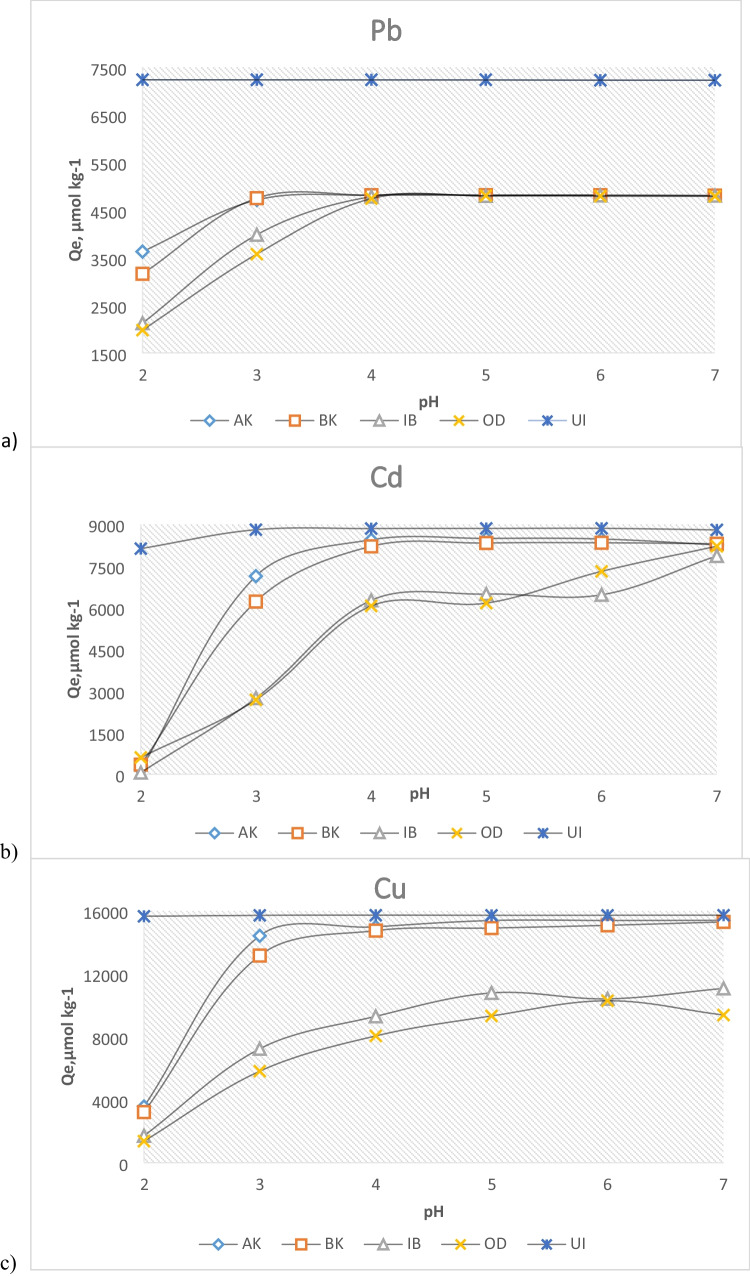
pH profiles of metal adsorption: **a** Pb, **b** Cd, and **c** Cu

Soil or solution pH is the most important parameter influencing metal-solution and soil-surface chemistry. Since most metal ions precipitate at pH above 8, which may even be lower in the presence of soil colloids, the pH dependency of the adsorption was studied between pH 2 and 7. As shown in Fig. 4, except for UI where adsorption was nearly 100 percent even at pH 2, metal adsorption was generally lowest at pH 2, increased very significantly from pH 2 to 3, and went to near completion between pH 3 and 4. According to Bradl et al. (2004) and Xie et al. (2018), adsorption of metal ions by soils increases from near zero to maximum over a relatively small pH range (i.e. pH-adsorption edge). As this study demonstrates, the pH range 2–4 is the pH-adsorption edge for most of the tested soil-metal systems.

There are several explanations for this correlation between pH and adsorbed amounts. Majorly, at low pH, the content of H^+^ is high, leading to soil mineral dissolution and release of ions such as Mg^2+^, Fe^2+^, and Al^3+^ which compete for the available adsorption sites with the metal ions being investigated. Secondly, most of the surface groups behave like Lewis acids or bases. At low pH, they become protonated, consequently producing positive surface charges which may weaken their abilities to form complexes with the heavy metal ions. Conversely, at high pH, the inorganic OH groups (silanol, aluminol, etc.) and the organic OH groups (COOH) become deprotonated (i.e. negatively charged), thus making it possible for the adsorbing cations to bond directly with them by ionic forces and surface coordinative mechanisms.

The near 100% adsorption of UI even at pH 2 was not in accordance with these arguments. However, one or a combination of the following reasons might be able to explain the phenomenon: (i) In agreement with the already established high adsorption capacity of this particular soil, the amount of added metal ions was essentially too low to saturate the available sorption sites; (ii) the soil has a high buffering capacity to resist appreciable mineral dissolution, thus making the amount of released Mg^2+^, Fe^2+^, and Al^3+^ insufficient to compete efficiently with the added metal ions for the abundant sorption sites; and (iii) comparably high amount of organic and inorganic mineral surfaces in this soil enhanced the formation of inner-sphere complexes thus enabling it to overcome the electrostatic repulsion created by the highly acidic condition (Blume et al. 2016).

### Adsorption kinetics

**Fig. 5:**
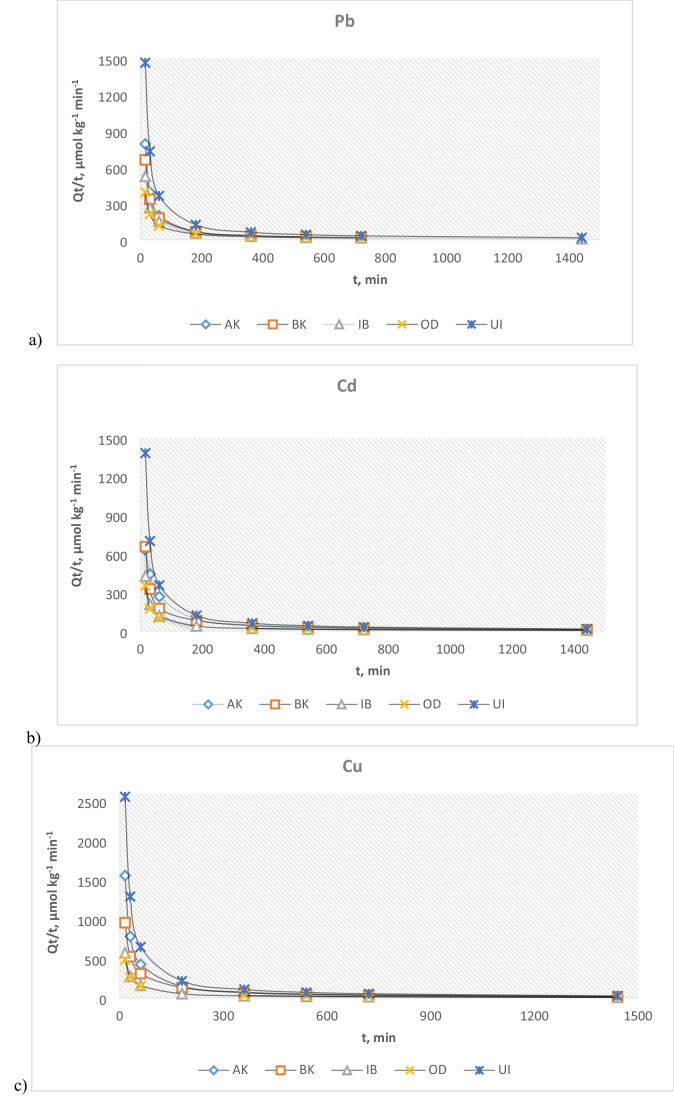
Kinetic profiles of **a** Pb, **b** Cd, and **c** Cu in the soil samples

Figure 5 shows the metal ion adsorption profile with time. As shown in these decline curves, the adsorption process can be classified into two stages: an early stage rapid adsorption (*t* = 0–60 min), followed by a slow rate-limiting second stage (*t* = 60–200 min), leading to an asymptote at long time. Although the data were fitted to both nonlinear PSO and TSKM equations, better fits were obtained with TSKM (*R*^2^ 0.80–0.98) than with PSO (*R*^2^ 0.70–0.87). Therefore, only the TSKM parameters are selectively presented (Table 3).

**Table 3:**
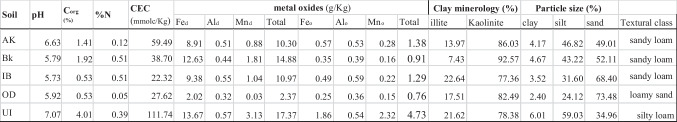
The two-stage kinetic model parameters

*Q*_e_ cal and *Q*_e_ exp (µmol kg^−1^) are the estimated and experimental adsorption quantity (respectively) of the metal ions per unit mass of the soil; *k*_1_ (min^−1^) is the fast sorption rate constant; and *k*_2_ (µmol kg^−1^ min^−0.5^) is the slow or diffusion rate constant

From Table 3, it could be observed that the model fits differ not only from soil to soil, but also from metal to metal, with Cu having the best overall fit. Despite the similarities in the *Q*_e_ cal for Pb and Cd, adsorption velocity, rated by *k*_1_, of Cd is lower than that of Pb and Cu. This is a further argument for higher mobility of Cd. According to McGrath and Cegarra (1992) and Bradl (2004), Cu shows relatively higher affinity for soil organic matter than the other metals. Therefore, relative contribution of the second adsorption stage, rated by *k*_2_, which is highest for Cu, might correlate with higher importance of organic matter for Cu adsorption. The trends of *Q*_e_ clearly agreed with the soil’s parameters, as already pointed out in the preceding section, and UI which has the highest of the measured soil parameters also gave the highest *k*_1_ for the three metals (1.4, 2.0, and 3.4 min^−1^, for Cd, Pb, and Cu, respectively). Nevertheless, *k*_1_ and *k*_2_ cannot be clearly explained by the soil’s physical–chemical attributes.

### ANFIS and MLR models

Ideally, the distribution coefficient (*K*_d_), rather than *Q*_e_, provides a better insight into the adsorptive behaviour of metals in soils and as such remains a valuable tool to assess and compare metal mobility and retention in soil systems (Alloway 1995; Covelo et al. 2004). However, the ANFIS and MLR models developed worked better for *Q*_e_ as the output vector, than for *K*_d_. While this may be connected to the reasons earlier stated, further, Souza et al. (2018) were also of the opinion that an intensive variable such as *Q*_e_ is a better choice as an output variable for adsorption modelling. It then follows that once *Q*_e_ can be accurately predicted, the corresponding *K*_d_ values can be calculated from Eq. 3, as previously stated. In this work, however, due to the high values of *Q*_e_ in µmol kg^−1^, and the need to scale the error matrices (MAE and RMSE) between 0 and 1, we used the log-transformed values of *Q*_e_ instead.

With the exhaustive search approach, we obtained a total of 255 models (i.e. 1 eight-vector model, 8 seven-vector models, 28 six-vector models, 56 five-vector models, 70 four-vector models, 56 three-vector models, 28 two-vector models, and 8 one-vector models) for each of ANFIS and MLR (both training and test systems). The models were assessed and compared based on their RMSE, MAE, and *R*^2^. While all the 255 ANFIS and 255 MLR models can be found as supplementary material to this paper, only few of the models are presented here (Table 4) to explain the major intricacies in our results. Nevertheless, the table captured the best of both MLR and ANFIS systems. It should be noted that the best model corresponds to the subset(s) of vectors that returned the lowest MAE and RMSE values, and the highest *R*^2^, for both training and test systems. Whereas more weight is usually placed on the test systems for overall decision on performance, good enough, the three performance indices in this work followed the same trends, in both training and test systems.

**Table 4:** Evaluation metrics of selected ANFIS and MLR models

Model	Input vectors	ANFIS										MLR									
		Test					Train					Test					Train				
		MAE		RMSE		*R*^2^-adj	MAE		RMSE		*R*^2^-adj	MAE		RMSE		*R*^2^-adj	MAE		RMSE		*R*^2^-adj
		Mean	s.d	Mean	s.d	Mean	Mean	s.d	Mean	s.d	Mean	Mean	s.d	Mean	s.d	Mean	Mean	s.d	Mean	s.d	Mean
M1	pH, *C*_o_, temp, *C*_org_, CEC, Fe, Mn, %clay	0.143	0.02	0.178	0.02	0.710	0.115	0.00	0.145	0.00	0.820	0.157	0.02	0.197	0.03	0.659	0.152	0.00	0.192	0.00	0.684
M2	pH, *C*_o_, *C*_org_, CEC, Fe, Mn, %clay	0.137	0.02	0.167	0.02	0.743	0.120	0.00	0.148	0.00	0.813	0.156	0.02	0.196	0.03	0.668	0.152	0.00	0.192	0.00	0.685
M3	pH, *C*_o_, *C*_org_, CEC, Fe, Mn	0.137	0.02	0.167	0.02	0.744	0.120	0.00	0.148	0.00	0.814	0.156	0.02	0.196	0.03	0.662	0.152	0.00	0.192	0.00	0.685
M4	pH, *C*_o_, *C*_org_, CEC, Fe, %clay	0.137	0.02	0.167	0.02	0.744	0.120	0.00	0.148	0.00	0.814	0.156	0.02	0.196	0.03	0.662	0.152	0.00	0.192	0.00	0.685
M5	pH, *C*_org_, CEC, Fe, Mn, %clay	0.257	0.03	0.313	0.04	0.171	0.245	0.00	0.305	0.00	0.211	0.255	0.04	0.312	0.04	0.180	0.251	0.00	0.309	0.00	0.188
M6	*C*_o_, *C*_org_, CEC, Fe, Mn, %clay	0.142	0.01	0.176	0.02	0.726	0.134	0.00	0.168	0.00	0.760	0.161	0.02	0.199	0.02	0.657	0.157	0.00	0.195	0.00	0.675
M7	pH, *C*_o_, *C*_org_, CEC, Fe	0.137	0.02	0.167	0.02	0.744	0.120	0.00	0.148	0.00	0.814	0.156	0.02	0.196	0.03	0.664	0.152	0.00	0.193	0.00	0.684
M8	pH, *C*_o_, CEC, Fe, %clay	0.137	0.02	0.167	0.02	0.744	0.120	0.00	0.148	0.00	0.814	0.155	0.02	0.195	0.03	0.667	0.152	0.00	0.193	0.00	0.685
M9	*C*_org_, CEC, Fe, Mn, %clay	0.266	0.04	0.321	0.04	0.120	0.263	0.00	0.319	0.00	0.139	0.266	0.04	0.321	0.04	0.134	0.263	0.00	0.319	0.00	0.139
M10	pH, *C*_o_, *C*_org_, Fe	0.135	0.02	0.164	0.02	0.753	0.120	0.00	0.148	0.00	0.814	0.155	0.03	0.196	0.03	0.664	0.152	0.00	0.194	0.00	0.682
M11	pH, *C*_o_, Fe, Mn	0.135	0.02	0.164	0.02	0.753	0.120	0.00	0.148	0.00	0.814	0.156	0.03	0.198	0.03	0.655	0.154	0.00	0.196	0.00	0.675
M12	pH, *C*_o_, Temp	0.144	0.02	0.176	0.03	0.725	0.134	0.00	0.166	0.00	0.767	0.172	0.03	0.213	0.03	0.609	0.170	0.00	0.212	0.00	0.623
M13	pH, *C*_o_, *C*_org_	0.131	0.02	0.160	0.02	0.769	0.121	0.00	0.149	0.00	0.813	0.154	0.02	0.195	0.03	0.667	0.152	0.00	0.194	0.00	0.682
M14	pH, *C*_o_, %clay	0.132	0.02	0.160	0.02	0.767	0.122	0.00	0.150	0.00	0.811	0.156	0.03	0.196	0.03	0.665	0.154	0.00	0.194	0.00	0.682
M15	pH, *C*_o_	0.140	0.02	0.170	0.03	0.743	0.136	0.00	0.168	0.00	0.764	0.172	0.03	0.213	0.03	0.611	0.170	0.00	0.212	0.00	0.622
M16	*C*_o_, *C*_org_	0.140	0.01	0.174	0.01	0.737	0.135	0.00	0.168	0.00	0.761	0.160	0.02	0.199	0.03	0.662	0.158	0.00	0.198	0.00	0.671
M17	*C*_o_, CEC	0.141	0.01	0.175	0.01	0.736	0.137	0.00	0.170	0.00	0.758	0.159	0.02	0.199	0.02	0.665	0.158	0.00	0.198	0.00	0.672
M18	pH	0.261	0.04	0.318	0.05	0.164	0.259	0.00	0.318	0.01	0.154	0.265	0.04	0.328	0.04	0.115	0.263	0.00	0.328	0.01	0.097
M19	*C*_o_	0.168	0.02	0.200	0.02	0.662	0.166	0.00	0.198	0.00	0.671	0.182	0.02	0.220	0.03	0.590	0.181	0.00	0.220	0.00	0.595
M20	Temp	0.276	0.04	0.341	0.05	0.058	0.274	0.00	0.340	0.01	0.033	0.277	0.05	0.341	0.05	0.039	0.276	0.01	0.342	0.01	0.020
M21	*C*_org_	0.266	0.04	0.321	0.04	0.145	0.263	0.00	0.319	0.00	0.148	0.268	0.04	0.322	0.04	0.131	0.266	0.00	0.322	0.00	0.129
M22	CEC	0.267	0.04	0.322	0.04	0.141	0.264	0.00	0.320	0.00	0.143	0.268	0.04	0.322	0.04	0.135	0.267	0.00	0.322	0.00	0.130
M23	Fe	0.269	0.04	0.324	0.04	0.122	0.267	0.00	0.323	0.00	0.124	0.271	0.04	0.329	0.04	0.100	0.270	0.00	0.329	0.00	0.095
M24	Mn	0.270	0.04	0.326	0.04	0.113	0.268	0.00	0.325	0.00	0.113	0.271	0.04	0.326	0.04	0.115	0.269	0.00	0.326	0.00	0.109
M25	%clay	0.268	0.04	0.323	0.04	0.128	0.266	0.00	0.322	0.00	0.132	0.267	0.04	0.322	0.04	0.134	0.266	0.00	0.322	0.00	0.130

As can be seen from Table 4, the best of ANFIS systems—M13 and M14 (both three-vector models)—returned *MAE*_test_ 0.131/0.132, *RMSE*_test_ 0.160, and *R*^2^_test_ 0.77, whereas their corresponding MLR models returned *MAE*_test_ 0.154/0.156, *RMSE*_test_ 0.195/0.196, and *R*^2^_test_ 0.67. Generally, the *R*^2^ values indicate that MLRs show greater deviation in fitting to the measured responses than their corresponding ANFIS. In addition, the MAE and RMSE for the ANFIS models are comparatively smaller than those of the corresponding MLRs, thus indicating that ANFIS is able to predict the adsorption with relatively lower error and uncertainty and/or higher accuracy. This confirms the previous arguments of Ghaedi et al. (2014), Rezaei et al. (2017), and Agbaogun et al. (2021) that because of the nonlinear nature of ANFIS, it has better predictive power than MLR.

Further, as earlier stated, the models were developed with combinations of both soil properties (*C*_org_, CEC, Fe, Mn, and %clay) and operational variables (*C*_o_, pH, and temperature). Since exhaustive search was used, these two groups also corresponded to two separate models, i.e. M9 (a subset exclusively of soil properties) and M12 (a subset exclusively of operational variables). While M9 (ANFIS) showed *MAE*_test_, *RMSE*_test_, and *R*^2^_test_ 0.266, 0.321, and 0.147, respectively, M12, on the other, showed *MAE*_test_, *RMSE*_test_, and *R*^2^_test_ 0.144, 0.176, and 0.73, respectively. This points out that whereas the adsorption conditions alone gave a very good model, explaining 73 percent of the residuals, the soil properties alone gave a very poor model, which explains only 16 percent of the residuals, and with considerably high uncertainty. Obviously, under given reaction conditions, the soil type is not as decisive as initially thought, and its influence on heavy metal ion distribution is comparably low. This agreed with the results obtained by Agbaogun et al. (2021) in the modelling of organic compounds (phenylurea herbicides) by ANFIS, using the same soils. According to Thiele and Leinweber (2001), it could be that the sorption equilibriums of both metal ions and organic compounds in these studies were underpinned by other soil properties not considered. Even *C*_o_ alone (M19) gave a better fit that than the combination of all soil parameters, explaining 66 percent of the residuals. pH (M18) explained 17 percent of the residuals, followed by *C*_org_ (M21) 15 percent. CEC, Fe, Mn, and %clay are almost equally weighted, explaining 12–14 percent, while temp explained only 6 percent of the residuals and returning the highest uncertainty indexes.

**Fig. 6:**
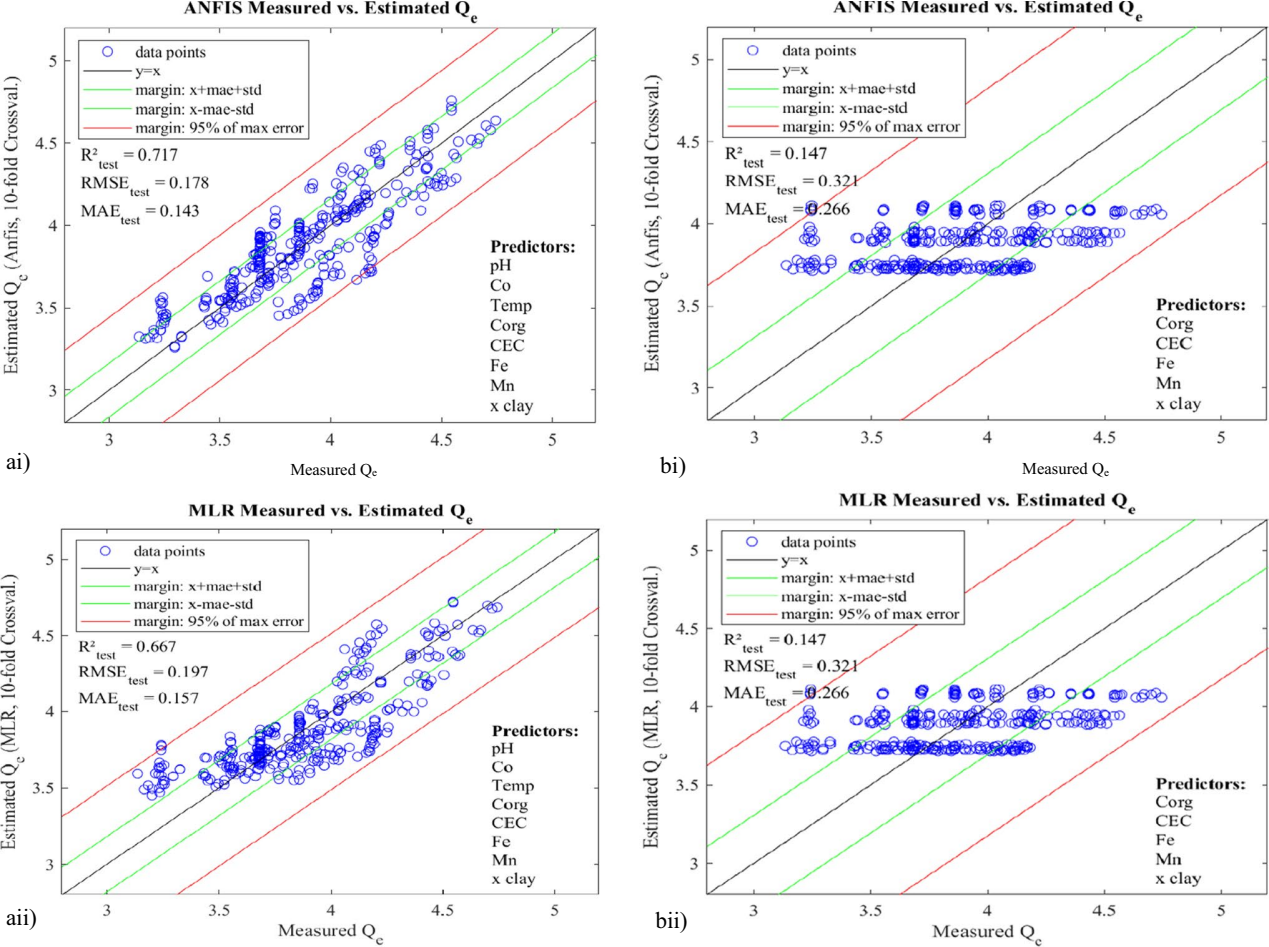

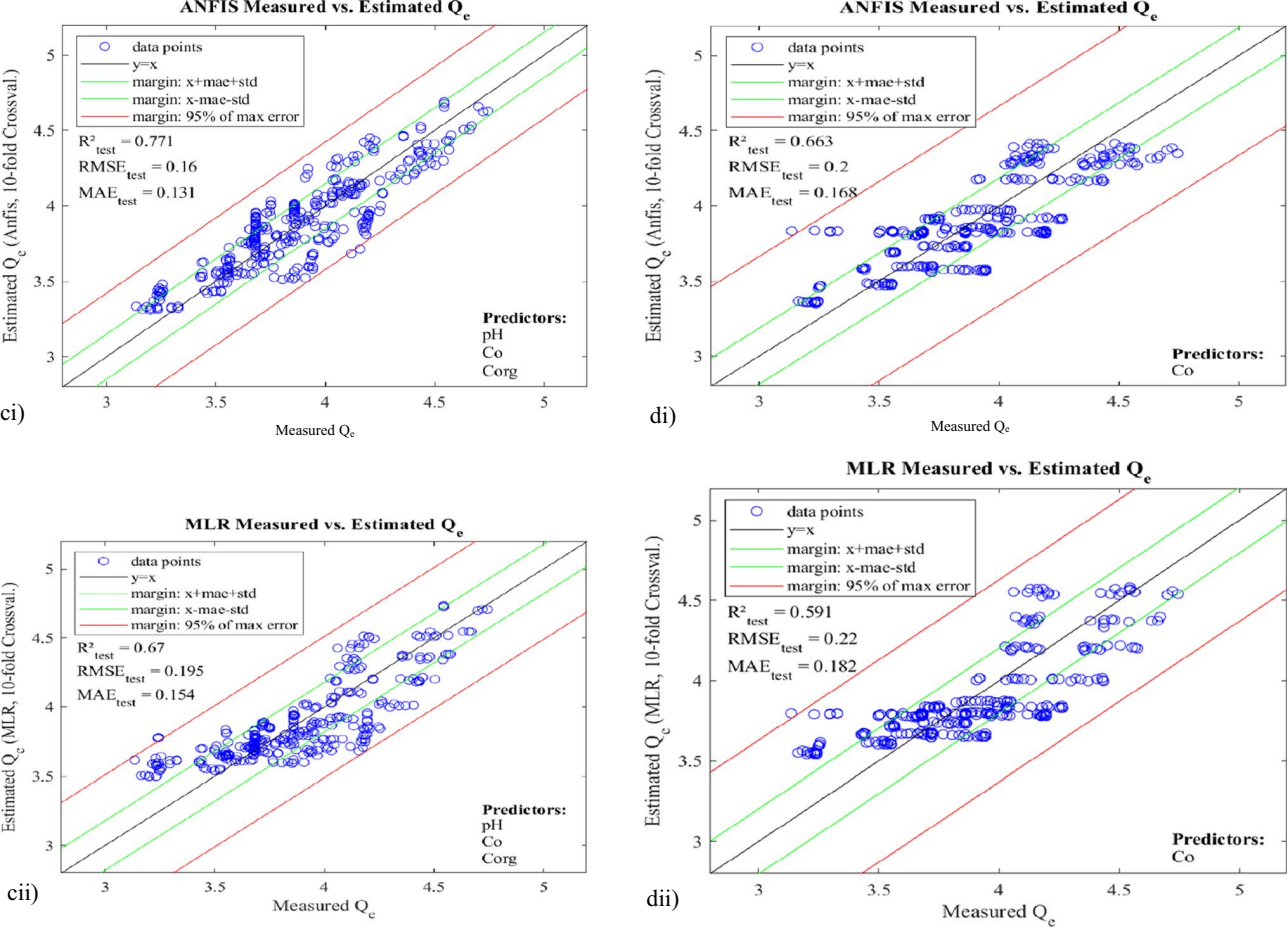
Scatterplots of *Q*_e_ (estimated) vs *Q*_e_ (experimental) µmol kg^−1^for **a** M1, **b** M9, **c** M13, and **d** M19, for both ANFIS and MLR test systems

Further, student’s *t* distribution was applied to calculate the scattering range of the predicted outputs vs actual outputs, at 95% (significance level), i.e. the confidence range in which 95% of all values are expected. This gave the opportunity to further analyse the effects of random errors in the models. Based on the scattering ranges and the distributions of points around the fitted lines (*y* = *x*) as shown in Fig. 6, one can gain further insights into the comparative advantages of ANFIS over MLR and also graphical relative performances of the selected models.

## Conclusion

The ability to adsorb or retain heavy metals has become one of soil’s major attributes, as it holds the potential to evaluate the environmental risks of metal ions. In this study, noncompetitive adsorption of Pb, Cu, and Cd onto tropical soils has been investigated. The sequence of affinity of the metal ions to soil, as indicated by their *Q*_m_*, is Cu > Pb > Cd. The lowest soil loading of Cd in this sequence is indicative of its higher environmental concern than Cu and Pb and explains why more of Cd could accumulate in the tissues of plants grown on sludge-treated plots than Cu or Pb (Berti and Jacobs 1996).

Several ANFIS and MLR models were developed to predict the equilibrium adsorption capacity (*Q*_e_) of these metals unto Nigerian soils, using the most influential variables such as soil/solution pH, initial metal ion concentration (*C*_o_), temperature, soil organic carbon (*C*_org_), CEC, amount of clay, and amorphous metal oxides (Fe and Mn). It can be inferred from the results of both models that under the given conditions, soil type is not as decisive as initially thought and its influence on metal ion distribution is low. This however throws up the need for further investigation, as some soil properties outside the ones investigated here could be decisive.

Further, the study shows that both ANFIS and MLR are suitable for predicting adsorption capacities. However, comparing their performances based on the three error metrics, ANFIS generally outperformed MLR. Conclusively, two ANFIS models, M13 and M14, were adjudged the best for the task of modelling the adsorption capacities of metal ions in soils. These models delivered overall three-vector combinations: pH, *C*_o_, and *C*_org_/%clay, and satisfied our search not only for the most accurate prediction but also for the smallest combination of input vectors to produce such prediction, with low uncertainty and high accuracy and interpretability trade-off. Given their ease of programmability, ANFIS models can be used as effective tools for in silico estimation of heavy metal partition or distribution equilibriums in untested soils, thus obviating the need for the expensive, laborious, and time-consuming field or laboratory investigations.

## Supplementary Information

Below is the link to the electronic supplementary material.

Supplementary file1 (XLSX 134 KB)

## Data Availability

The data leading to this manuscript as well as the Matlab codes for the elaborated ANFIS and MLR models are available.
